# The detection of African trypanosomes in goats reared in tsetse infested villages of Eastern Zambia

**DOI:** 10.1007/s11250-022-03367-5

**Published:** 2022-11-03

**Authors:** Gloria M. Mulenga, Bruce Gummow

**Affiliations:** 1 College of Public Health Medical and Veterinary Sciences, James Cook University, Townsville, QLD 4814 Australia; 2 Department of Veterinary Services, Kakumbi Tsetse and Trypanosomiasis Research Station, P.O Box 70, 10101 Mfuwe, Zambia; 3 Faculty of Veterinary Science, University of Pretoria, Pretoria, 0028 South Africa

**Keywords:** Trypanosomiasis, Goats, Prevalence, Zambia

## Abstract

Control programmes for African animal trypanosomiasis (AAT) in livestock have been mainly focused on cattle with very little focus on goats, an important reservoir for the disease. Using the polymerase chain reaction (PCR), this study investigated trypanosome infection in village goats in Mambwe, a rural District in Eastern Zambia**.** Filter paper blood spots were collected from 326 goats and tested for infection with *Trypanosoma congolense*, *Trypanosoma vivax* and *Trypanosoma brucei s.l*. using ribosomal RNA internal transcribed spacers (ITS)-PCR. The frequency of trypanosomes from the sampled goats was 4.6% (95% CI = 2.3–6.8). Results indicated significantly high infections with *Trypanosoma vivax* (4.0%; 95% CI = 1.9–6.1) than *T. congolense* (0.6%; 95% CI =  − 0.2 to 1.5)*,* and *T. brucei* (0.0%), *P* = 0.04. Findings show the circulation of trypanosomes that causes AAT in goats and that they may pose serious threats to not only goats but also to other livestock reared alongside goats.

## Introduction

Tsetse transmitted trypanosomiasis is an important disease in sub-Saharan Africa and has continued to threaten food security (FAO, 2018; Franco et al., 2022). While crop farming is a major economic activity in Zambia, livestock farming is also practiced by a number of small-scale farmers who depend on livestock rearing for their livelihood (Lysholm et al., 2020). Trypanosomiasis in small ruminants has increasingly become important especially with an increase in human encroachment into tsetse and wildlife interface areas (Kebede et al., 2009). For remote rural districts like Mambwe, small ruminants play an economically important role for small-scale farmers who are unable to keep large animals such as cattle. Apart from providing meat, milk, manure, and skin for famers, goats provide liquid assets and are also a source of household savings (Kebede et al., 2009; Von Wissmann et al., 2011). The control and management of both human and animal trypanosomiasis through treatment in livestock reservoirs has been evaluated using cattle but has not been explored in small ruminants including goats. Previously goats have been considered to be tolerant to trypanosome infection and that they play a minimum role in the transmission of trypanosomiasis and have thus not been targeted for control programmes (Hamill et al., 2017). This study was, therefore, conducted to investigate *T. brucei*, *T. congolense* and *T. vivax* infections in village goats using internal transcribed spacer-polymerase chain reaction (ITS-PCR) due to the ability of the test to detect mixed trypanosomes from field samples. Primers ITS1 CF and ITS1 BR, have been evaluated for use in a universal diagnostic test for all pathogenic trypanosomes because of its highly conserved flanking regions and size variability among trypanosomes species and subgroups (Desquesnes et al., 2001; Njiru et al., 2005).

## Materials and methods

Using an estimated prevalence of 60% (Ruiz et al., 2015), error margin of 5%, 326 goats were sampled from 193 livestock-owning small-scale farmers of Mambwe District, Eastern Zambia. Livestock farmers were drawn from four villages located about 50 km from each other: Nsefu, Katemo, Chikowa and Ncheka. Using a micro-capillary tube, about 200 µl of blood was drawn from each selected animal after puncturing the ear veins of the animals with a blood lancet. From each goat sample, blood spots were applied on Whatman® No. 1 filter paper (GE Healthcare) and air-dried before packing in a zip locked storage bag containing silica gel. DNA from stored blood spots was extracted using the buffer technique as described by Morrison et al. (2007). PCR was undertaken in 25 µl reaction mixtures containing primers ITS1 CF (5ʹ-CCGGAAGTTCACCGATATTG-3ʹ) and ITS1 BR (5ʹ-TTGCTGCGTTCTTCAACGAA-3ʹ), One Taq 2X master mix (New England BioLabs, Ipswich, MA, USA), nuclease free water and 5 µl of extracted DNA sample, all reagents procured from Inqaba Biotec, Pretoria, South Africa (Radwanska et al., 2002; Njiru et al., 2005; Mulenga et al., 2021).

## Results and discussion

Demographics of trypanosome infection as recorded from the four study sites were as follows: (Nsefu = 80 sampled, 3 infected; Katemo = 80 sample, 0 infected; Chikowa = 81 sampled, 11 infected, Ncheka = 85 sampled, 1 infected).

The frequency of *Trypanosoma vivax* as detected by ITS-PCR was 4.0% (13/326) and that of *T*. *congolense* was 0.6% (2/326). No *T*. *brucei* nor mixed infections were reported in this study. Sampled goats were significantly more infected with *T*. *vivax* infections than *T. congolense* (*t* test = 2.87, *P* value = 0.04) (Table 1).

**Table 1:** Proportion of goats sampled in the Luangwa valley, Eastern Zambia with trypanosomes in the year 2019

Trypanosome species	No. positive	Sample prevalence %	Confidence Interval at 95%
*T. congolense*	2	0.6	− 0.2 to 1.5
*T. vivax*	13	4.0	1.9 to 6.1
*T. brucei*	0	0	0
*Mixed*	0	0	0
**Total**	**15**	**4.6**	**2.3–6.8**

Our results indicate that trypanosomiasis is prevalent and widely spread among goat farmers in Mambwe District of the Eastern Province of Zambia. Most livestock farmers in Eastern Zambia, rear goats, and other small ruminants alongside cattle with livestock treatments, exclusively carried out in cattle. This poses a great threat to livestock health and production (Laohasinnarong et al., 2015). Results obtained in this study showed similar trypanosome infection levels (< 5%) as those obtained by Kebede et al. (2009) and Simukoko et al. (2007)), which were much lower than findings obtained from Nyimba et al. (2015), 23.7%. This may be attributed to difference in sensitivities of the methods used and trypanosomiasis challenge in the study area. However, the use of ITS-PCR as a universal PCR-based test adds value to the collection of epidemiological data on trypanosomiasis, while easing the cost of running several PCRs, especially in the endemic zones of Africa (Njiru et al., 2005; Von Wissmann et al., 2011).

Despite our study reporting no cases of *T. brucei* from the goats sampled, indicating that *T. brucei* was not circulating in the goats sampled. Our findings were consistent with observations made by Kebede et al. (2009) and Van den Bossche et al. (2010), but disagreed with findings from other studies where *T. brucei* was found to be highly prevalent (Von Wissmann et al., 2011; Hassan-Kadle et al., 2020). The absence of *T. brucei* in our study may have been attributed to the inability of the sample collection technique i.e., filter paper, to preserve enough DNA to be detected by PCR. Filter paper, however, inhibits ITS-PCR, making it less accurate compared to when DNA is extracted directly from whole blood samples (Ahmed et al., 2013). The frequency in trypanosome species, *T. congolense* and *T. vivax* distribution were similar with other findings (Kebede et al., 2009; Von Wissmann et al., 2011; Maganga et al., 2020), where goats were found to be highly prevalent in *T. vivax* as compared to cattle which is highly prevalent in *T. congolense* (Hassan-Kadle et al., 2020; Mulenga et al., 2021). In livestock, *T. congolense* and *T. vivax* are the most prevalent under natural infections, while *T. brucei* is the least prevalent (Van den Bossche and Delespaux, 2011; Maganga et al., 2020). The frequency of trypanosomes in goats indicates that goats are important reservoirs of trypanosomes that causes AAT and should be considered when undertaking AAT treatment control programmes in livestock.

## Data Availability

Not applicable.
